# Endoscopic Approach to Laryngeal Schwannoma: A Case Report

**DOI:** 10.1007/s12070-025-05341-7

**Published:** 2025-02-13

**Authors:** Akanksha Garg, Anmol Kath, Prince Handa, Akshit Vermani

**Affiliations:** 1 Department of Head and Neck Oncology, Homi Baba Cancer Centre Sangrur, Punjab, 148001 India; 2 Department of Oral and Maxillofacial surgery, Guru Nanak Dev Dental College, Sunam, 148028 India

**Keywords:** Laryngeal schwannoma, Surgical treatment, Endoscopic approach, Neurogenic neoplasm

## Abstract

Laryngeal schwannoma is an uncommon clinical condition. This study reports the case of 78-year-old female with history of voice change. The tumour was removed by transoral endoscopic approach. On follow up the patient was symptom free. The aim of this report is to advert comprehension about this rare disease.

## Introduction

Neurogenic neoplasms of the head and neck are uncommon. Schwannomas are uncommon benign nerve sheath tumours that originate from the neural crest’s Schwann cells. They account for approximately 5% of all head and neck tumours. Schwannomas, most of which are benign, can develop from any peripheral, cranial, or autonomic nerve.

Approximately 25–45% of schwannomas occur in the head and neck [1, 2]. Malignant transformation is rarely seen in laryngeal schwannoma.

The current study presents the case of a schwannoma arising in posterior commissure area in a 78 -year-old female.

## Case Report

A 78-year-old female presented to opd with complaints of voice change since one year with a history of trigeminal neuralgia. No neck nodes were palpated. An indirect laryngoscopy examination reveals mucosal hypertrophy over posterior commissure area with bilateral mobile vocal cords. On direct laryngoscopy a well-defined nodular firm swelling with smooth margins were present over posterior commissure with no ulceration. The biopsy taken was confirmed to be schwannoma. Patient defaulted for one year and reported back with progressive change in voice and no frank stridor. On fibreoptic laryngoscopy examination nodular mass seen in posterior commissure area with mild thickening over true vocal cords with no restriction in mobility.

**Fig. 1:**
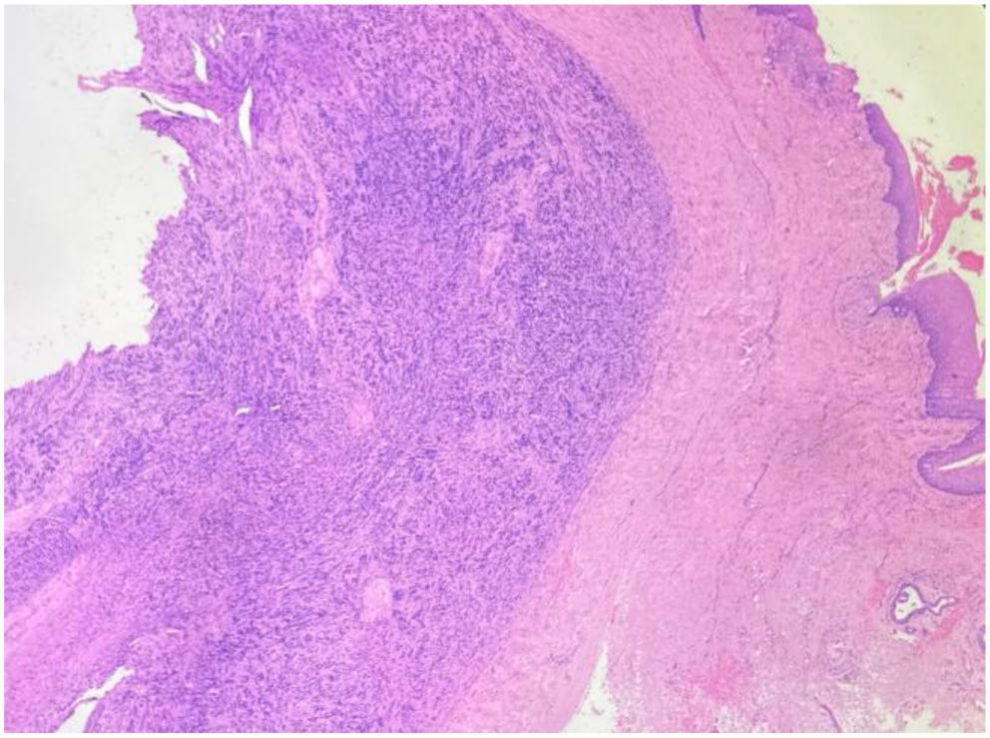
H&E Stained Section Revealing a Well Circumscribed Lesion Composed of Spindle Cells in the Subepithelial Tissue ( H&E, 100x)

**Fig. 2:**
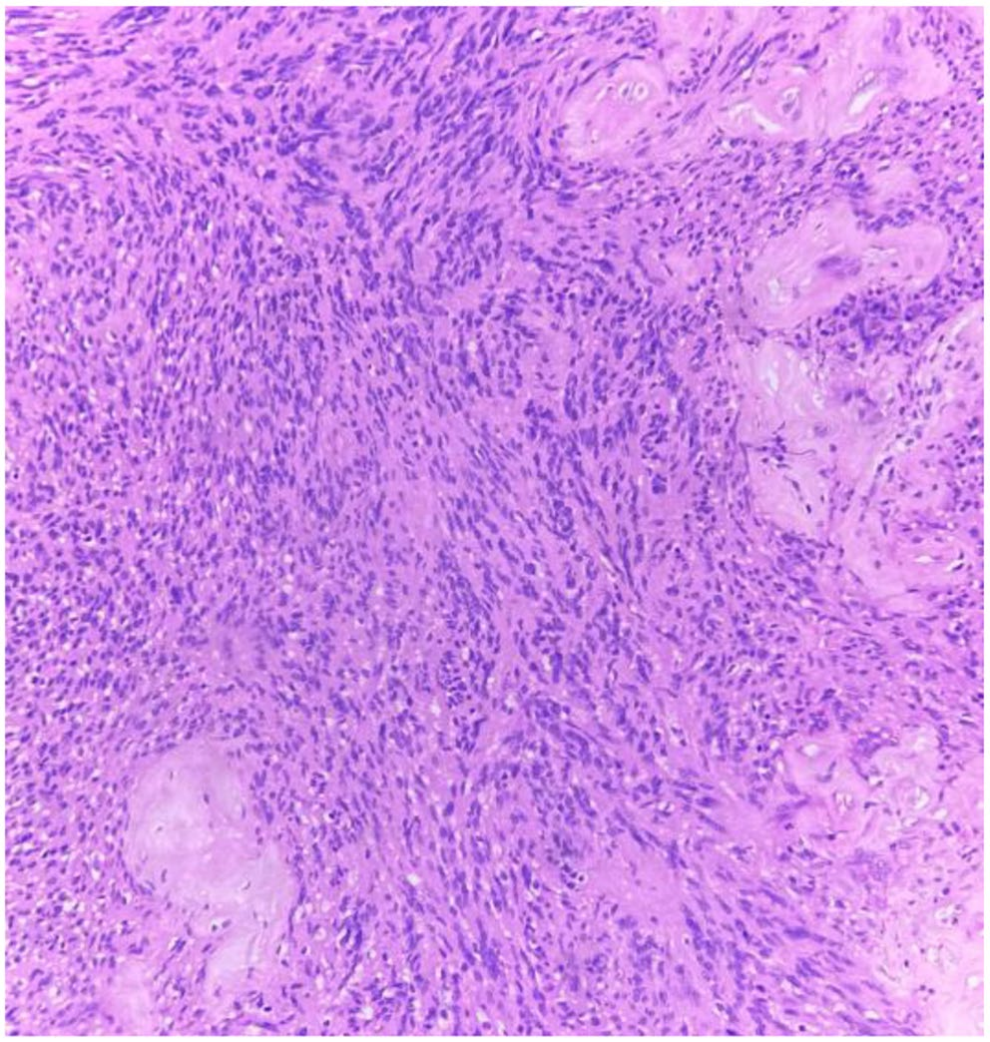
High Power view Showing Tumour Cells Aligned in Palisaded rows Along with Hyalinised Blood Vessels (H&E, 400 x)

**Fig. 3:**
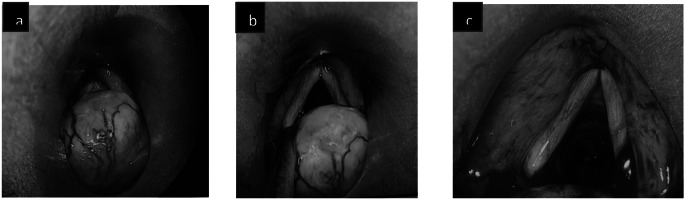
(**a** to **b**) Intraoperative Image Showing Laryngeal Schwannoma (**c**) Complete Excision of the Lesion

**Fig. 4:**
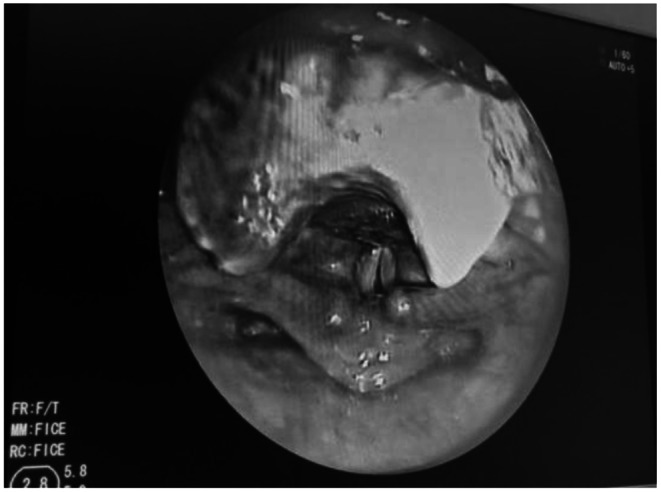
Post-Operative view Following no Residual Lesion

For further evaluation CT neck was done which showed soft tissue thickening 1.9 × 1.6 cm mass noted involving posterior commissure and bilateral aryepiglottic fold. The treatment planned was endoscopic assisted laryngeal schwannoma excision. Following successful intubation endoscopic assisted excision of laryngeal schwannoma with tracheostomy was performed with the help of cold instruments. The operative site was thoroughly examined for any remaining residue. Following extubation, the vocal cords were examined for mobility and found to be normal. The excised specimen was sent for histopathological examination (Figs. 1, 2). The patient was on follow up for three weeks postoperatively. Patient improved symptomatically and on examination with fibreoptic laryngoscopy normal movement of vocal cords were noted with no residual disease (Figs. 3, 4).

## Disscussion

Schwannomas are encapsulated tumours that are benign and slow growing; develop from schwann cells of any nerve, whether peripheral, cranial, or autonomic. Commonly, they are parapharyngeal or intracranial [3]. Of all the schwannomas (25–45%) tumours located in parapharyngeal spaces being the most common among head and neck region [2, 4]. Laryngeal schwannomas are of rare clinical presentation. The aryepiglottic fold is the most prevalent anatomical site, followed by the arytenoids, ventricular fold, vocal cords [3]. Rarely subglottic region is involved which is often fatal. The schwannoma is presumed to originate in the internal branch of the superior laryngeal nerve. Smaller nerve fibres in the laryngeal submucosa are often the other site of its origin [3]. Laryngeal schwannomas can be found in both age group being more common in females in their 5th-6th decade of life [2, 5]. Malignant transformation of laryngeal schwannoma is extremely rare.

Laryngeal schwannoma patients often exhibit a variety of symptoms pertaining to the tumour’s location and mass effect [6]. Symptoms may include globus sensation, dysphagia, odynophagia, dysphonia/hoarseness, dyspnea with exertion, or stridor [2, 7, 8]. Patients with slow-growing schwannoma may experience non-specific symptoms for months or years before being diagnosed [2]. 

Radiological investigations (X-ray neck, CT scan, and MRI neck) and clinical examination by laryngoscopy (direct or indirect) can aid in the diagnosis.

Laryngoscopy reveals laryngeal schwannoma as round submucosal swelling, regardless of the subsite. CT and MRI are helpful in determining the nature and extent of a lesion, with MRI providing better soft tissue delineation.

Most authors describe the disease as a well-defined, hypodense submucosal mass with no signs of infiltrative growth as seen in CT scans whereas in case of MRI scanning in T1-weighted images the lesion is expected to be isointense to slightly hyperintense and in T2, the lesion is hyperintense [9, 10]. 

Ultrasound used in certain cases reveal slightly heterogeneous mass with a regular contour [11]. The larynx and lesion’s rear edge, however, can be hard to see on ultrasonography [7]. Therefore, ultrasound should not be used for diagnostic purposes in these patients.

Although the clinical and radiological characteristics are peculiar but not distinctive to schwannoma. Various differential diagnosis can be made to schwannoma which include - neurofibroma, laryngocele, laryngeal cyst, adenomas, and malignant tumour [2]. Therefore, a histopathological examination is necessary to make a definitive diagnosis.

Histological diagnoses can be obtained through various methods such as FNAC, incisional biopsy, or excisional biopsy. However histologically schwannoma and neurofibroma or another subtype of benign nerve sheath neoplasms can be difficult to differentiate. It’s crucial to distinguish between the two because neurofibroma has a higher risk of recurrence and malignant potential (10%) [8].

Schwannoma is diagnosed based on Enger and Weiss histologic criteria: the presence of a capsule, Antoni A and/or B areas, S-100 protein positivity [2, 7]. 

To effectively manage schwannoma, it’s important to distinguish between pedunculated and nonpedunculated types. The presence of the peduncle facilitates complete surgical excision.

Conversely, the nonpedunculated schwannomas underwent distinct treatment methods based on characteristics, such as tumour location and size. Surgical resection of the mass using various approaches has been the preferred treatment method. Since schwannomas are radioresistant hence radiation therapy is ineffective [8]. For effective surgical treatment, complete excision with minimal damage to the uninvolved areas of the larynx is required.

For tumours that are small and superficial or pedunculated with good Endo laryngeal exposure, endoscopic excision with or without laser is the preferred option [7]. For large tumours, an external approach may provide optimal exposure for complete excision along the capsule [7]. Compared to the endoscopic approach, the open approach increases the risk of postoperative vocal cord paralysis/hypomobility.

## Conclusion

Even though neurogenic tumours are a rare entity, they need to be managed as they can cause life-threatening airway obstruction. For resection of small or pedunculated lesions transoral endoscopic approach is the preferred treatment option with minimal chance of complications.
